# Guillain-Barré Syndrome with asystole requiring permanent pacemaker: a case report

**DOI:** 10.1186/1752-1947-3-5

**Published:** 2009-01-06

**Authors:** Mehul B Patel, Sandeep K Goyal, Sujeeth R Punnam, Khyati Pandya, Vipin Khetarpal, Ranjan K Thakur

**Affiliations:** 1 Thoracic and Cardiovascular Institute, Sparrow Health System, Michigan State, University, Lansing, MI, USA; 2 Department of Internal Medicine, Michigan State University, East Lansing, MI, USA

## Abstract

**Introduction:**

Guillain-Barré syndrome is an acute demyelinating disorder of the peripheral nervous system that results from an aberrant immune response directed at peripheral nerves. Autonomic abnormalities in Guillain-Barré syndrome are usually transient and reversible. We present a case of Guillain-Barré syndrome requiring a permanent pacemaker in view of persistent symptomatic bradyarrhythmia.

**Case Presentation:**

An 18-year-old Caucasian female presented with bilateral lower limb paraesthesias followed by bilateral progressive leg weakness and difficulty in walking. She reported an episode of an upper respiratory tract infection 3 weeks prior to the onset of her neurological symptoms. Diagnosis of Guillain-Barré syndrome was considered and a lumbar puncture was performed. Cerebrospinal fluid revealed albuminocytologic dissociation (increased protein but normal white blood cell count) suggestive of Guillain-Barré syndrome and hence an intravenous immunoglobulin G infusion was started. Within 48 hours, she progressed to complete flaccid quadriparesis with involvement of respiratory muscles requiring mechanical ventilatory support. Whist in the intensive care unit, she developed multiple episodes of bradycardia and asystole requiring a temporary pacemaker. In view of the persistent requirement for the temporary pacemaker for more than 5 days, she received a permanent pacemaker. She returned for follow-up three months after discharge with an intermittent need for ventricular pacing.

**Conclusion:**

Guillain-Barré syndrome can result in permanent damage to the cardiac conduction system. Patients with multiple episodes of bradycardia and asystole in the setting of Guillain-Barré syndrome should be evaluated and considered as potential candidates for permanent pacemaker implantation.

## Introduction

Autonomic neuropathy is an important complication of Guillain-Barré syndrome (GBS), seen in about 60% cases. It is common in young adults, presents with more severe syndromes, and accounts for the mortality in severely affected individuals. Cardiac autonomic impairment in GBS includes labile hypertension, orthostatic hypotension, and a wide range of cardiac arrhythmias including sinus tachycardia, serious bradyarrhythmias and asystole. These manifestations occur primarily from either an under activity or an excessive activity of the sympathetic or parasympathetic pathways. We report a case of Guillain-Barré syndrome requiring permanent pacemaker for severe bradycardia.

## Case Presentation

An 18-year-old Caucasian female presented with bilateral lower limb paraesthesias followed by increasing leg weakness and difficulty in walking over a period of 2 days. She reported an episode of an upper respiratory tract infection 3 weeks prior to the onset of her neurological symptoms. Past, personal and social history was unremarkable. Clinical examination revealed decreased muscle strength in all extremities associated with hypotonia and areflexia.

A diagnosis of Guillain-Barré Syndrome (GBS) was considered and a lumbar puncture was performed. Cerebrospinal fluid (CSF) revealed albuminocytologic dissociation (elevated protein with normal white blood cell count in CSF) suggestive of GBS. She was started on intravenous immunoglobulin G, but within 48 hours, she progressed to complete flaccid quadriparesis with involvement of respiratory muscles and required mechanical ventilatory support.

**Figure 1:**
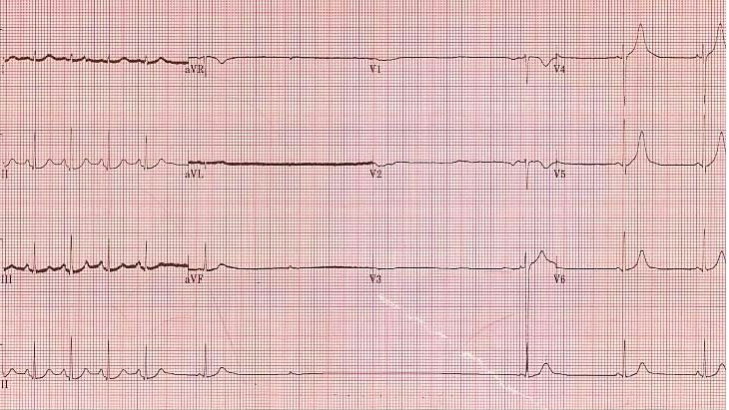
**A 12-lead ECG showing a 4.2 second pause**.

On day 12, a cardiac electrophysiology consultation was requested for bradycardia and multiple episodes of asystole. These episodes occurred spontaneously, unrelated to tracheobronchial suctioning, blood drawing or any other intervention. The longest observed pause was 12 seconds. Electrolyte profile was normal and oxygenation was satisfactory. The result of her 12-lead ECG is shown in Figure 1.

In view of the multiple episodes of bradycardia and asystole the decision was made to insert a pacemaker. The patient initially received a temporary pacemaker. However, due to an anticipated prolonged clinical course and the potential for recurrent bradycardia and asystole, an INSIGNIA Ultra DR dual chamber permanent pacemaker was implanted after 5 days. The pacemaker was initially programmed to VVI mode at 40 beats per minute to prevent pacing as much as possible. The pacemaker mode was switched to DDDR at the time of discharge because of lack of spontaneous sinus node activity. A paced rhythm was present 18 days post implant, suggesting occurrence of intermittent bradycardia.

The patient returned to our office for a routine pacemaker check 3 months after implantation. The pacemaker check revealed that she was in paced rhythm for most of the time in this period. Seventy percent was atrial paced ventricular sensed rhythm with a set lower rate of 40 beats per min. Twenty percent was atrial paced ventricular paced rhythm with a set AV delay of 220 msec. Only for ten percent of the time was she in atrial sensed ventricular sensed rhythm. This may indicate prolonged influence on the autonomic tone even after complete somatic recovery and likely justifies the need for a permanent pacemaker. As far as choice of pacemaker mode is concerned, our patient received a DDDR mode. However, as the 3 month follow up interrogation showed ventricular pacing of <40%, a Managed Ventricular Pacing or the AAIsafeR would have also been a good pacemaker mode option in retrospect.

## Discussion

Guillain-Barré Syndrome (GBS) is an acute demyelinating disorder of the peripheral nervous system that results from an aberrant immune response directed at peripheral nerves. Average annual incidence is 1.7 per 100,000 [1]. A typical GBS patient presents with rapidly ascending symmetrical weakness, which may progress to respiratory failure in 30% of patients [2].

Autonomic dysfunction has been described in GBS and was noted in as many as 66% of patients in one study [3]. Pathologic studies of the autonomic nervous system in GBS may demonstrate edema and inflammation of autonomic ganglia and destruction of peripheral ganglion cells. Chromatolysis, mononuclear cell infiltration, and nodules of Nageotte can be found within sympathetic ganglia [4]. Autoantibodies against gangliosides are often present, such as with anti-GM1 antibodies. Clinical manifestations of dysautonomia can range from seemingly innocuous profuse perspiration to life threatening arrhythmias. Sir William Osler described a patient with GBS who died of "paralysis of the heart" [5]. Autonomic disturbance most commonly presents as sinus tachycardia, labile hypertension and postural hypotension. However, sinus bradycardia, asystole, supraventricular tachycardia, junctional tachycardia and ventricular tachycardia have also been reported. The risk of dysautonomia is higher in patients with quadriplegia, respiratory failure or bulbar involvement [6]. Recent studies have indicated that serious bradyarrhythmias were observed even in less severely affected patients [7].

Bradyarrhythmias occur in up to 50% of patients with severe GBS and are due to parasympathetic overactivity [8]. Episodes of sinus arrest can happen during endotracheal suctioning in patients on ventilators, but can also happen spontaneously (as in our patient). It results from a malfunction of afferent baroreceptor reflex. Ropper et al [9] postulated that afferent baroreflex failure causes labile blood pressure and release of sympathetic efferents leading to catecholamine excess. This, in turn, sensitizes left ventricular stretch receptors and other nociceptors causing a compensatory reflex bradycardia. Manifestations of both sympathetic and parasympathetic excess may be seen in the same patient.

Parasympathetic overactivity may be intermittent, may cause serious bradyarrhythmias ranging from bradycardia to asystole, and may account for a significant number of deaths in GBS patients [4]. It is commonly believed that marked bradyarrhythmias occur only in severely affected patients, especially in patients requiring mechanical ventilation [10,11]. However, they have also been reported in less critically ill patients who do not require mechanical ventilation [12]. Flachenecker et al have described the eyeball pressure test and the 24-hour heart rate power spectrum for predicting which patients with GBS will develop clinically significant bradycardia[12,13].

Review of the literature regarding management of bradyarrhythmias associated with GBS shows a lack of uniform opinion. The treatment approach has ranged from the use of isoproterenol and atropine, to insertion of a temporary or permanent pacemaker [14,15].

## Conclusion

Bradyarrhythmias and asystole can be a complicating factor in GBS with autonomic involvement, requiring careful monitoring in the ICU setting. Physicians should be vigilant about the presence of these abnormalities in patients with GBS. Early involvement of an electrophysiology team in the care of such patients is important. A permanent pacemaker may be a reasonable intervention if a protracted recovery is expected.

## Abbreviations

GBS: Guillain-Barré Syndrome; ECG: electrocardiogram; PPM: permanent pacemaker; VVI: single lead pacemaker in the ventricle that is set at a fixed rate: it is inhibited by a detected ventricular beat; CSF: cerebrospinal fluid; DDDR: dual chamber rate adaptive pacemaker; AAIsafeR: a pacemaker mode to prevent unnecessary ventricular pacing

## Consent

Written informed consent was obtained from the patient for publication of this case report and accompanying images. A copy of the written consent is available for review by the Editor-in-Chief of this journal.

## Competing interests

The authors declare that they have no competing interests.

## Authors' contributions

MBP and SRP participated in the collection of data and patient care. SKG and KP in the preparation of the manuscript. VK provided collection of data and editing of the manuscript, RKT participated in patient care, final revision of the manuscript and guidance. All authors read and approved the final manuscript
